# Application of Nalbuphine in Trigeminal Ganglion Pulse Radiofrequency Surgery in Patients with Postherpetic Neuralgia

**DOI:** 10.1155/2021/6623112

**Published:** 2021-03-01

**Authors:** Song Wen, Qiu-Xia Xiao, Zhao-Qiong Zhu, Li Chen, Ying Li, Qi-Hua Ran, Song Cao, Liu-Lin Xiong

**Affiliations:** ^1^Department of Anesthesiology, Department of Pain Medicine, Affiliated Hospital of Zunyi Medical University, Zunyi 563000, China; ^2^Southwest Medical University, Luzhou 646000, China; ^3^Institute of Neurological Disease, West China Hospital, Sichuan University, Chengdu 610041, China

## Abstract

This study aimed to explore the application value of nalbuphine in pulsed radiofrequency operation of trigeminal ganglion in patients with postherpetic neuralgia (PHN). Thirty patients with PHN were randomly divided into the nalbuphine (Nalbu) group and ketorolac tromethamine (KT) group and received CT-guided pulsed radiofrequency surgery on trigeminal ganglion. The numeric rating scale (NRS) scores of patients were recorded at preoperative, intraoperative, and postoperative time points, before going to bed, and the next morning after the operation. In addition, the number of breakthrough pain before operation and within 24 hours after operation, the incidence of nausea and vomiting within 24 hours after surgery, and the patient's sleep quality before and on the day after surgery were evaluated. The outcome data demonstrated that patients treated with nalbuphine had lower NRS scores after the pulse radiofrequency operation during and after the pulse radiofrequency operation compared to those with KT. In addition, nalbuphine effectively decreased the number of breakthrough pain, reduced the occurrence of nausea and vomiting after surgery, and improved the sleep quality. In conclusion, intramuscular injection of nalbuphine 30 min before trigeminal ganglion pulse radiofrequency surgery can be conducive to pain relief and improve the postoperative comfort of patients, providing an effective alternative for the alleviation of PHN in clinic.

## 1. Introduction

Postherpetic neuralgia (PHN) refers to the pain of herpes zoster that lasts 1 month or more after the rash healed [1], representing as the most common complication of herpes zoster [2]. PHN is a kind of stubborn neuropathic pain [3], with which patients may experience different types of pain including a steady burning pain and a sudden stabbing pain or stimulus-evoked pain (allodynia) [2, 4]. The annual incidence of herpes zoster reaches about 3 to 5% [5], and the reported risk of developing PHN in patients with herpes zoster varied widely ranging from 5% to more than 30% [6], exhibiting an increasing tendency with age [5, 7]. The emotion, sleep quality, and daily life of some patients with PHN are moderately to severely disturbed [8]. The more severe the pain is, the greater the impact on vitality, sleep, and overall quality of life [9]. It is worth noting that the family members of patients are also prone to fatigue, stress, insomnia, and emotional distress [10]. The conventional drug treatment takes suboptimal efficacy, and the mechanism of PHN remains not fully understood. Research studies have revealed that the underlying mechanism may involve peripheral sensitization, central sensitization, inflammatory reaction, and differentiation [11]. Increasing attention has been paid to the trigeminal nerve which is challenging due to its special location and severity of pain.

PHN that cannot be controlled by drugs can be treated with minimally invasive interventional surgery to alleviate the pain. Minimally invasive interventional therapy refers to the technique of inserting instruments or drugs into the diseased tissue with minimal trauma under the guidance of imaging, followed by physical, mechanical, or chemical treatment on it [4]. Pulse radiofrequency is a neuromodulation therapy in which pulsed radiofrequency current is usually used for treatment. It is safe and minimally invasive, with few side effects, exhibiting great potential and clinical application value for the treatment of neuropathic pain in patients who are refractory to conservative treatments [12]. It is now clear that pulsed radiofrequency technology can effectively relieve neuralgia after herpes zoster and trigeminal neuralgia [13, 14]. Pulsed radiofrequency could effectively alleviate pain and improve the life quality of patients without damaging effect on nerve fiber structure. Additionally, such complications as hypoesthesia, soreness, burning pain, and motor nerve injury are less common [15]. Trigeminal pulse radiofrequency surgery has a good effect on PHN in the head and face area [16, 17]. As a result of the need for sensory testing during the pulsed radiofrequency process, the original pain area can be replicated to confirm that the radiofrequency needle tip is adjacent to the corresponding ganglion area. Thus, severe pain will be induced, and analgesics need to be given before surgery to reduce intraoperative pain. Nonsteroidal analgesics are commonly used as perioperative pain control drugs, but such drugs are contraindicated in patients with gastrointestinal bleeding and peptic ulcer and have a greater impact on renal function. Therefore, it is urgent to find effective analgesic drugs with small side effects.

Nalbuphine hydrochloride injection has both *μ*-antagonist and *κ*-agonist activities with strong analgesic effect, which can reduce *μ* receptor-mediated related complications, such as respiratory depression, itching, nausea, and vomiting [18]. Nalbuphine can be administered by intravenous infusion, intramuscular injection, subcutaneous injection, or intrathecal injection. The intravenous administration takes effect within 2-3 minutes, and the subcutaneous and intramuscular injection takes effect within 15 minutes; the peak effect reaches 30 minutes. It has certain advantages in day surgery. Studies have shown that nalbuphine assisting nonsteroidal anti-inflammatory drugs can significantly enhance postoperative analgesia and reduce postoperative complications [19], which have a good clinical application prospect. Ketorolac has an anti-inflammatory and analgesic effect by inhibiting the biosynthesis of prostaglandins, prostacyclin, and thromboxane; however, whose postoperative analgesic effect was found to be not quite satisfactory when used alone [20]. Relative to commonly used nonsteroidal intraoperative analgesic ketorolac tromethamine, nalbuphine provides analgesia without the undesirable side effects of the pure agonists and provides superior postoperative analgesia by reducing inflammatory and oxidative stress [21]. However, the differential efficacy of these two drugs remains obscure in alleviating PHN. Hence, in this study, we compared the analgesic effect of nalbuphine and ketorolac tromethamine in patients with pain in the trigeminal innervation area secondary to PHN after trigeminal ganglion pulse radiofrequency surgery and exploited the advantages of nalbuphine in the pulsed radiofrequency operation of the trigeminal ganglion for treating PHN.

## 2. Materials and Methods

### 2.1. Case Collection and Grouping

**Figure 1:**
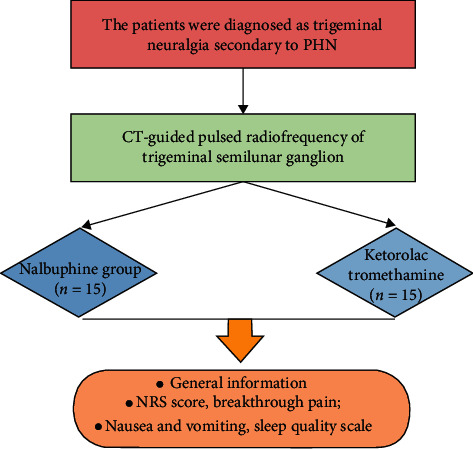
The flowchart of the study. PHN, postherpetic neuralgia; CT, computer tomography; NRS, numeric rating scale.

This study was reviewed and approved by the Medical Ethics Committee of the Affiliated Hospital of Zunyi Medical University. Thirty cases of PHN patients with V2, V3, or V2/V3 trigeminal neuralgia and planned to undergo the trigeminal/semilunar ganglion pulsed radiofrequency surgery were included from January 2018 to September 2020. Meanwhile, basic information of patient was collected (Figure 1). The patients were randomly divided into two groups, including the nalbuphine (Nalbu) group and ketorolac tromethamine (KT) group. In the Nalbu group, patients received intramuscular injection of 0.2 mg/kg nalbuphine 30 min before surgery; in the KT group, patients received intramuscular injection of 60 mg ketorolac tromethamine 1 h before surgery.

### 2.2. Case Inclusion and Exclusion Criteria

Inclusion criteria: patients were diagnosed with PHN of the V2, V3, or V2/V3 trigeminal nerve by two or more chief physicians at the same time; patients suffer severe pain (NRS score ≥8), which cannot be relieved by medicine. The PHN patients were aware of this study and signed informed consent. Patients suffered PHN lasts for more than one month after the rash has healed [22], and PHN-induced trigeminal neuralgia is distributed in the trigeminal nerve innervation area. Patients suffered various types of trigeminal neuralgia, a steady burning pain, a sudden stabbing pain, or stimulus-evoked pain (allodynia) in the V2 or/and V3 distributed area, but without Ramsay Hunt syndrome, e.g., facial paralysis, tinnitus, ear pain, and hearing loss.

Exclusion criteria: patients underwent astrointestinal bleeding or gastrointestinal ulcer; patients with chronic gastritis, coagulation dysfunction, a history of mental illness, or unable to cooperate with surgery; patients with insufficiency of the heart, kidneys, and lungs; patients with respiratory or digestive diseases or infections; and patients who had contraindications to puncture and who refused invasive treatment were excluded.

### 2.3. Pulsed Radiofrequency Operation

**Figure 2:**
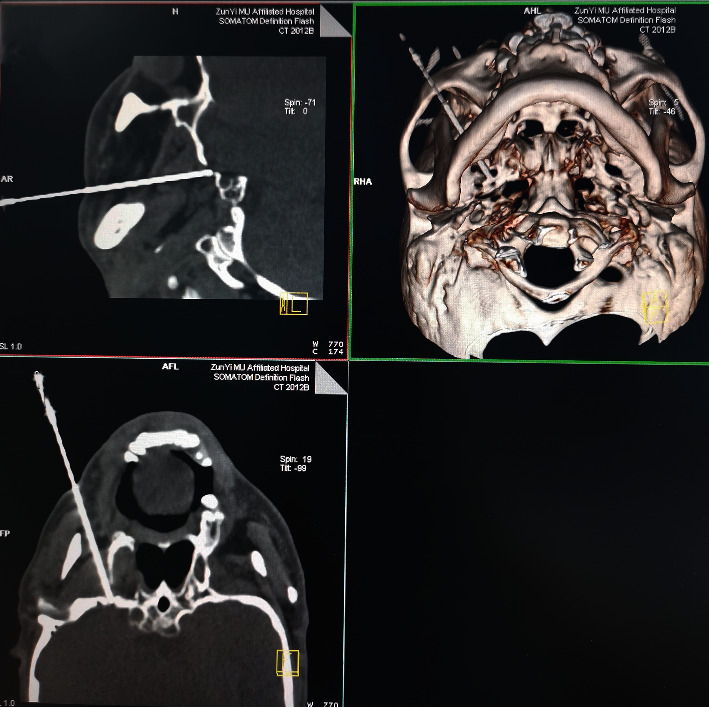
The images of pulsed radiofrequency operation.

After entering the computerized tomography (CT) room, the electrocardiograph (ECG), noninvasive blood pressure, and pulse oximetry of the patients in the supine position were routinely monitored, and the peripheral venous access was established. The operation area was disinfected and covered with sterile towels. After local anesthesia with 3 ml of 1% lidocaine, a radiofrequency trocar (22G, 10 cm, 5 mm, Inomed, 240102, Germany) was punctured passing 2 cm outside the affected side of the mouth under the guidance of CT. Three-dimensional reconstruction of the bone window was used to determine the needle direction and depth until the needle tip enters the trigeminal ganglion in the ipsilateral foramen ovale (Figure 2). The sensory test was performed after the needle tip reached the foramen ovale. A radiofrequency thermocoagulator (Beiqi Medical, R2000B, Beijing) with a frequency of 50 Hz, a voltage of 0.1–0.3 V, and a resistance of 300–500 Ω was used for sensory testing to replicate the pain in the original pain area so as to confirm the tip of the radiofrequency needle and simulate the corresponding ganglion area. After that, a pulsed radiofrequency procedure (42°C, 2 Hz, 10 min) was conducted. After the operation, the radiofrequency electrode and radiofrequency needle were removed, and the puncture point was attached. The patient was observed and returned to the ward without discomfort.

### 2.4. Indicator Collection

The vital signs of the patient were closely monitored during the operation. The numeric rating scale (NRS) was used to evaluate the degree of pain. The NRS scores were collected before, during, and at the end of the operation, before going to bed, and the next morning after the operation. In addition, the total number of breakthrough pain was recorded, and the sleep quality before operation and within 24 hours after surgery was evaluated with the Self-Rating Scale of Sleep (SRSS). The higher the score, the more serious the sleep problem [23]. The incidence of nausea and vomiting were recorded within 24 hours after surgery.

### 2.5. Statistical Analysis

SPSS 22.0 statistical software was used to analyze the data. Data were analyzed by the independent sample *t*-test and expressed as mean ± standard deviation (SD). Sex ratio between two groups was analyzed with the chi-square test. *P* < 0.05 indicated that the difference was statistically significant.

## 3. Results

### 3.1. Comparison of Basic Information

**Figure 3:**
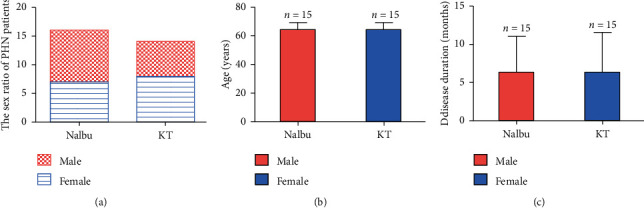
General information of patients between two groups. (a) The sex ratio of PHN patients; (b) age of patients; (c) disease duration of patients. Nalbu, nalbuphine; KT, ketorolac tromethamine. *n* = 15 for each group.

Among 15 patients in the Nalbu group, there were 7 males and 8 females, 50–79 years old, with an average age of 64.0 ± 5.1 years old. The course of disease was 1–20 months, with an average course of 6.3 ± 4.8 months. In the KT group, there were 15 patients, including 9 males and 6 females, 50–75 years old, with an average age of 63.7 ± 6.3 years old. The course of disease ranged from 1 to 20 months, with an average course of 6.6 ± 5.1 months. There was no statistical significance in sex, age, and course of disease between the two groups (*P* > 0.05, Figures 3(a)–3(c)).

### 3.2. NRS Scores between Two Groups

**Figure 4:**
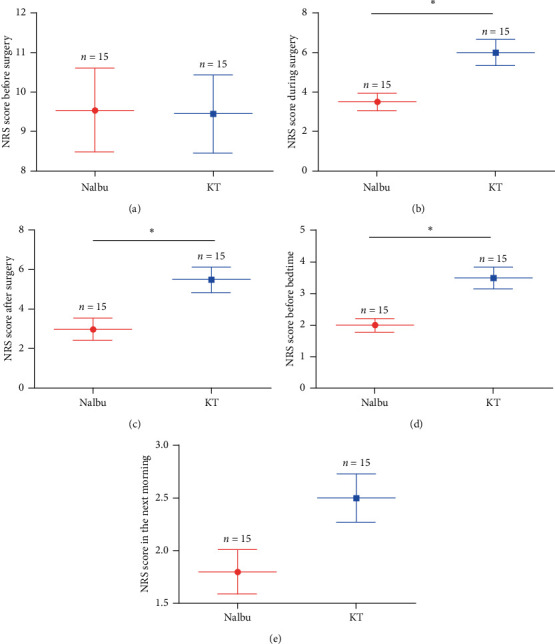
The NRS scores of patients between two groups. (a) NRS score before surgery; (b) NRS score during surgery; (c) NRS score after surgery; (d) NRS score before bedtime; (e) NRS score in the next morning. NRS, numerical rating scale; Nalbu, nalbuphine; KT, ketorolac tromethamine. *n* = 15 for each group, ^*∗*^*P* < 0.05.

There was no statistical difference in the preoperative NRS scores between the two groups (*P* > 0.05, Figure 4(a)). During the operation, at the end of the operation, and before bedtime after the operation, the NRS scores of patients in the Nalbu group who received pulsed radiofrequency were lower than those in the KT group, and the difference was statistically different (*P* < 0.05, Figures 4(b)–4(d)). The NRS scores of patients exhibited no statistical difference at the next morning after the operation between Nalbu and KT groups (*P* > 0.05, Figure 4(e)).

### 3.3. Nalbuphine Decreased the Number of Breakthrough Pain

**Figure 5:**
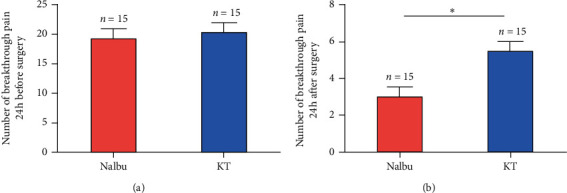
Number of breakthrough pain. (a) Number of breakthrough pain 24 h before surgery; (b) number of breakthrough pain 24 h after surgery. Nalbu, nalbuphine; KT, ketorolac tromethamine; (h) hours. *n* = 15 for each group, ^*∗*^*P* < 0.05.

There was no significant difference in the number of breakthrough pain between the two groups before surgery (*P* > 0.05, Figure 5(a)); however, the number of postoperative breakthrough pain in the Nalbu group was significantly less than that in the KT group, and the difference was statistically significant (*P* < 0.05, Figure 5(b)).

### 3.4. Nalbuphine Decreased Nausea and Vomiting

**Figure 6:**
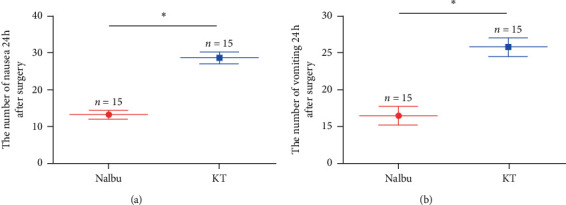
The number of nausea and vomiting 24 h after surgery. (a) The number of nausea 24 h after surgery; (b) the number of vomiting 24 h after surgery. Nalbu, nalbuphine; KT, ketorolac tromethamine; (h) hours. *n* = 15 for each group, ^*∗*^*P* < 0.05.

Patients in the Nalbu group had fewer nausea attacks than those in the KT group at 24 hours after surgery (*P* < 0.05, Figure 6(a)). At the same time, the number of vomiting attacks in the Nalbu group was also reduced relative to that in the KT group 24 hours after surgery (*P* < 0.05, Figure 6(b)).

### 3.5. Nalbuphine Improved Sleep Quality

**Figure 7:**
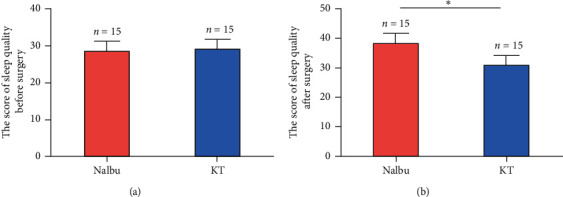
The scores from the Self-Rating Scale of Sleep (SRSS) between two groups. (a) The score of sleep quality before surgery; (b) the score of sleep quality after surgery. Nalbu, nalbuphine; KT, ketorolac tromethamine. *n* = 15 for each group, ^*∗*^*P* < 0.05.

No statistical difference was shown in the preoperative sleep scores between the two groups (*P* > 0.05, Figure 7(a)), while the sleep scores of patients in the Nalbu group were higher than those in the KT group 24 hours after surgery, and the difference was statistically significant (*P* < 0.05, Figure 7(b)).

## 4. Discussion

This study explored the application value of nalbuphine in pulsed radiofrequency surgery on the trigeminal ganglion of patients with PHN. The results demonstrated that nalbuphine could effectively reduce the pain score and the occurrence of adverse reactions after pulsed radiofrequency and, meanwhile, significantly improve the sleep quality in patients obsessed with PHN.

### 4.1. Application of CT-Guided Pulsed Radiofrequency Operation on Trigeminal Ganglion

In this study, CT-guided pulsed radiofrequency was performed on trigeminal ganglion to attenuate the PHN. Herpetic neuralgia is a true type of neuropathic pain, and its occurrence in the trigeminal nerve is a challenge in the medical domain because of its special location and severity of pain. Radiofrequency is a reliable method for the treatment of neuropathic pain in patients who are refractory to conservative treatment. Currently, radiofrequency on the trigeminal ganglion is an effective method for the treatment of primary trigeminal neuralgia [24, 25]. Pulsed radiofrequency is a neuromodulation technology that uses electrical impulses to stimulate the nerves that produce pain in a timely manner, adjusting the nerve function in a feedback manner, or producing numbness to cover the painful area and to achieve the effect of treating pain [16]. Pulsed radiofrequency can affect sensory nerve ATP metabolism and ion channel function and continuously and reversibly inhibit C fiber excitatory afferents, thereby blocking the pain transmission of related nerves [25]. Studies have confirmed that pulsed radiofrequency has no damaging effect on the structure of nerve fibers. It can not only relieve pain but also rarely cause sensory loss and motor nerve function damage after treatment, which is increasingly being used in the treatment of PHN [5, 26], trigeminal neuralgia [27], musculoskeletal pain conditions [28], and chronic cervical and lumbosacral pain [29]. Increasing evidence showed that CT-guided radiofrequency can effectively treat the facial neuralgia, such as trigeminal neuralgia [30]. The CT-guided pulsed radiofrequency is one of the classic representative methods and has been widely used for trigeminal neuralgia treatment [31, 32]. In the present study, the pulsed radiofrequency under the guidance of CT greatly increased puncture efficiency and safety during operation. The pulsed radiofrequency surgery significantly reduced the pain intensity in the two groups of PHN patients (NRS before vs after the surgery: 9.54 vs 3.0 in the Nalbu group; 9.45 vs 5.5 in the KT group, Figure 4). The minimal invasiveness and effectiveness of the pulsed radiofrequency will enable it to be widely used in clinical treatment.

### 4.2. The Analgesic Advantages of Nalbuphine

Here, 0.2 mg/kg nalbuphine was injected intramuscularly 30 min before pulsed radiofrequency surgery to alleviate the discomforts of patients during and after surgery. Nalbuphine is an opioid agonist-antagonist of the phenanthrene series which was synthesized in an attempt to provide analgesia without the undesirable side effects of the pure agonists. Its analgesic and antipruritic effects are mediated via actions on the *μ* and *κ*-receptors, which has been indicated for mild to moderate pain. Its analgesic effect is equivalent to morphine [33], which is particularly effective for visceral pain and has a sedative effect [34–36]. Nalbuphine also seems to provide superior postoperative analgesia by reducing inflammatory and oxidative stress [37]. The analgesic effect is rapid (5–10 min) and enduring, which is suitable for prehospital first aid, balanced anesthesia or conscious anesthesia, postoperative analgesia, and treatment of chronic pain [38–41]. In addition, a previous research reported that the response of nalbuphine combined with naloxone significantly alleviated the pain of 3 patients with refractory neuropathic trigeminal neuralgia [42]. Consistently, our results demonstrated that nalbuphine treatment significantly lowered the NRS scores of patients during the operation, at the end of the operation, and before bedtime after the operation relative to ketorolac tromethamine treatment, suggesting that nalbuphine could alleviate the perioperative pain and may be a new treatment for neuropathic pain.

### 4.3. Nalbuphine Reduces Postoperative Complications

To further validate the efficacy of nalbuphine in patients undergoing the pulsed radiofrequency, the number of postoperative breakthrough pain and the frequency of nausea and vomiting were recorded, and the sleep quality of patients was also evaluated. Nalbuphine exerts a strong, fast, and enduring analgesic and sedative effect, with almost no cardiovascular adverse reactions [43], light respiratory depression, and a capping effect [44, 45]. Many studies have confirmed the efficacy of intravenous nalbuphine in the treatment of itching caused by opioids, which can reduce patients' nausea and vomiting, and reverse adverse reactions such as respiratory depression [19, 43]. Intravenous administration of low-dose nalbuphine (5 *μ*g/kg/h) in patients with epidural morphine not only maintains the analgesic effect but also reduces the occurrence of itching [46]. Among elderly patients undergoing total hip arthroplasty under spinal anesthesia, the analgesic effect of the nalbuphine was faster than the morphine, and nalbuphine decreased the occurrence of respiratory depression, nausea, vomiting, and itching more effectively than morphine [47]. In addition, nalbuphine suppresses pruritus in a dose-dependent manner and is expected to be used to treat pruritus in uremic patients without the need for dosage adjustment [48]. A recent study reported that the use of opioid receptor agonist-antagonist nalbuphine successfully reversed the adverse effects of urinary retention caused by epidural morphine on the bladder [49]. At the same time, it will not reverse the postoperative analgesic effect or induce other significant adverse reactions [50, 51]. Accumulative evidence demonstrated that nalbuphine is more effective in preventing itching caused by intrathecal morphine after cesarean section [52]. Consistent with abovementioned findings, our work revealed that in comparison with the effect of ketorolac tromethamine, the nalbuphine application significantly diminished the number of postoperative breakthrough pain, decreased the frequency of nausea and vomiting, and greatly improved the sleep quality of patients who underwent pulsed radiofrequency surgery to alleviate PHN.

## 5. Conclusions and Limitations

Collectively, nalbuphine administration before surgery can alleviate the pain during and after the trigeminal/semilunar ganglion pulse radiofrequency and effectively reduce the postoperative adverse reactions. These findings confirmed the important role of nalbuphine exerting a stronger analgesic and sedative effect than ketorolac tromethamine, providing an alternative for clinical multimodal analgesia. In our study, the mean age (64.0 ± 5.1) of patients is much lower than that found in other publications [53]. This might be attributed to the sampling errors and the small number of patients involved in the present study. The bigger number of included patients is essential for the future studies.

## Data Availability

The data of this work are available from the corresponding author on reasonable request.

## References

[1] Rowbotham M. C., Davies P. S., Fields H. L. (1995). Topical lidocaine gel relieves postherpetic neuralgia. *Annals of Neurology*.

[2] Johnson R. W., Rice A. S. C. (2014). Postherpetic neuralgia. *New England Journal of Medicine*.

[3] Ma L. L., Cao S., Li Y. (2018). Advances of lidocaine intravenous infusion in the treatment of neuropathic pain. *Journal of Zunyi Medical University*.

[4] Gan E. Y., Tian E. A. L., Tey H. L. (2013). Management of herpes zoster and post-herpetic neuralgia. *American Journal of Clinical Dermatology*.

[5] Kawai K., Gebremeskel B. G., Acosta C. J. (2014). Systematic review of incidence and complications of herpes zoster: toward a global perspective. *BMJ Open*.

[6] Sampathkumar P., Drage L. A., Martin D. P. (2009). Herpes zoster (shingles) and postherpetic neuralgia. *Mayo Clinic Proceedings*.

[7] Opstelten W., Van Wijck A. J., Van Essen G. A. (2004). The PINE study: rationale and design of a randomised comparison of epidural injection of local anaesthetics and steroids versus care-as-usual to prevent postherpetic neuralgia in the elderly. *BMC Anesthesiology*.

[8] Oster G., Harding G., Dukes E., Edelsberg J., Cleary P. D. (2005). Pain, medication use, and health-related quality of life in older persons with postherpetic neuralgia: results from a population-based survey. *The Journal of Pain*.

[9] Mauskopf J., Austin R., Dix L., Berzon R. (1994). The nottingham health profile as a measure of quality of life in zoster patients: convergent and discriminant validity. *Quality of Life Research*.

[10] Weinke T., Glogger A., Bertrand I., Lukas K. (2014). The societal impact of herpes zoster and postherpetic neuralgia on patients, life partners, and children of patients in Germany. *Scientific World Journal*.

[11] Johnson R. W., Wasner G., Saddier P., Baron R. (2007). Postherpetic neuralgia: epidemiology, pathophysiology and management. *Expert Review of Neurotherapeutics*.

[12] Vanneste T., Van Lantschoot A., Van Boxem K., Van Zundert J. (2017). Pulsed radiofrequency in chronic pain. *Current Opinion in Anaesthesiology*.

[13] Kim K., Jo D., Kim E. (2017). Pulsed radiofrequency to the dorsal root ganglion in acute herpes zoster and postherpetic neuralgia. *Pain Physician*.

[14] Ding Y., Li H., Hong T., Zhao R., Yao P., Zhao G. (2019). Efficacy and safety of computed tomography-Guided pulsed radiofrequency modulation of thoracic dorsal root ganglion on herpes zoster neuralgia. *Neuromodulation: Technology at the Neural Interface*.

[15] Ma K., Fan Y. H., Jin Y. (2013). Efficacy of pulsed radiofrequency in the treatment of thoracic postherpetic neuralgia from the angulus costae: a randomized, double-blinded, controlled trial. *Pain Physician*.

[16] Ding Y. Y., Hong T., Li H. X., Yao P., Zhao G. Y. (2019). Efficacy of CT guided pulsed radiofrequency treatment for trigeminal postherpetic neuralgia. *Frontiers in Neuroscience*.

[17] Wan C., Dong D. S., Song T. (2019). High-voltage, long-duration pulsed radiofrequency on gasserian ganglion improves acute/subacute zoster-related trigeminal neuralgia: a randomized, double-blinded, controlled trial. *Pain Physician*.

[18] Zeng Z., Lu J. H., Shu C. (2015). A comparision of nalbuphine with morphine for analgesic effects and safety: meta-analysis of randomized controlled trials. *Science Reports*.

[19] Mao Y., Cao Y. Y., Mei B. (2018). Efficacy of nalbuphine with flurbiprofen on multimodal analgesia with transverse abdominis plane block in elderly patients undergoing open gastrointestinal surgery: a randomized, controlled, double-blinded trial. *Pain Reserach and Management*.

[20] Parke T. J., Millett S., Old S., Goodwin A. P. L., Rice A. S. C. (1995). Ketorolac for early postoperative analgesia. *Journal of Clinical Anesthesia*.

[21] Jaffe J. H., Martin W. R. (1990). Opioid analgesics and antagonists. *The Pharmacological Basis of Therapeutics*.

[22] Yang F., Yu S., Fan B. (2019). The epidemiology of herpes zoster and postherpetic neuralgia in China: results from a cross-sectional study. *Pain and Therapy*.

[23] Yamaguchi N., Matsubara S., Momonoi F., Morikawa K., Takeyama M., Maeda Y. (1998). An attempt of radar chart expression of a self-rating scale for sleep disturbance. *Psychiatry and Clinical Neurosciences*.

[24] Zhang H., Ni H., Liu S., Xie K. (2020). Supraorbital nerve radiofrequency for severe neuralgia caused by herpes zoster ophthalmicus. *Pain Reserach and Management*.

[25] Erdine S., Bilir A., Cosman E. R. (2009). Ultrastructural changes in axons following exposure to pulsed radiofrequency fields. *Pain Practice*.

[26] Van Hecke O., Austin S. K., Khan R. A., Smith B. H., Torrance N. (2014). Neuropathic pain in the general population: a systematic review of epidemiological studies. *Pain*.

[27] Thapa D., Ahuja V., Dass C., Verma P. (2015). Management of refractory trigeminal neuralgia using extended duration pulsed radiofrequency application. *Pain Physician*.

[28] Wu Y.-T., Ho C.-W., Chen Y.-L., Li T.-Y., Lee K.-C., Chen L.-C. (2014). Ultrasound-guided pulsed radiofrequency stimulation of the suprascapular nerve for adhesive capsulitis. *Anesthesia & Analgesia*.

[29] Van Zundert J., Patijn J., Kessels A., Lamé I., van Suijlekom H., Van Kleef M. (2007). Pulsed radiofrequency adjacent to the cervical dorsal root ganglion in chronic cervical radicular pain: a double blind sham controlled randomized clinical trial. *Pain*.

[30] Tang Y. Z., Jin D., Li X. Y., Lai G. H., Li N., Ni J. X. (2014). Repeated CT-guided percutaneous radiofrequency thermocoagulation for recurrent trigeminal neuralgia. *European Neurology*.

[31] Lai G.-H., Tang Y.-Z., Wang X.-P., Qin H.-J., Ni J.-X. (2015). CT-guided percutaneous radiofrequency thermocoagulation for recurrent trigeminal neuralgia after microvascular decompression. *Medicine*.

[32] Han Z.-X., Chen M.-J., Zhang W.-J., Chai Y., Zhang W.-H. (2020). Tooth-supported personalized template-assisted foramen ovale puncture system for trigeminal neuralgia treatment. *Journal of Clinical Neuroscience*.

[33] Zacny J. P., Conley K., Marks S. (1997). Comparing the subjective, psychomotor and physiological effects of intravenous nalbuphine and morphine in healthy volunteers. *The Journal of Pharmacology and Experimental Therapeutics*.

[34] Pierre J.-M. R. (2004). Peripheral kappa-opioid agonists for visceral pain. *British Journal of Pharmacology*.

[35] Schmauss C., Doherty C., Yaksh T. L. (1982). The analgetic effects of an intrathecally administered partial opiate agonist, nalbuphine hydrochloride. *European Journal of Pharmacology*.

[36] Tiwari A. K., Tomar G. S., Agrawal J. (2013). Intrathecal bupivacaine in comparison with a combination of nalbuphine and bupivacaine for subarachnoid block. *American Journal of Therapeutics*.

[37] Xi M. Y., Li S. S., Zhang C., Zhang L., Wang T., Yu C. (2020). Nalbuphine for analgesia after orthognathic surgery and its effect on postoperative inflammatory and oxidative stress: a randomized double-blind controlled trial. *Journal of Oral and Maxillofacial Surgery*.

[38] Woollard M., Jones T., Pitt K., Vetter N. (2002). Hitting them where it hurts? Low dose nalbuphine therapy. *Emergency Medicine Journal*.

[39] Naaz S., Shukla U., Srivastava S., Ozair E., Asghar A. (2017). A comparative study of analgesic effect of intrathecal nalbuphine and fentanyl as adjuvant in lower limb orthopaedic surgery. *Journal of Clinical and Diagnostic Research: JCDR*.

[40] Nazir N., Jain S. (2017). Randomized controlled trial for evaluating the analgesic effect of nalbuphine as an adjuvant to bupivacaine in supraclavicular block under ultrasound guidance. *Anesthesia Essays and Researches*.

[41] Chatrath V., Attri J. P., Bala A., Khetarpal R., Ahuja D., Kaur S. (2015). Epidural nalbuphine for postoperative analgesia in orthopedic surgery. *Anesthesia Essays and Researches*.

[42] Schmidt B. L., Gear R. W., Levine J. D. (2003). Response of neuropathic trigeminal pain to the combination of low-dose nalbuphine plus naloxone in humans. *Neuroscience Letters*.

[43] Lake C. L., Duckworth E. N., Difazio C. A., Durbin C. G., Magruder M. R. (1982). Cardiovascular effects of nalbuphine in patients with coronary or valvular heart disease. *Anesthesiology*.

[44] Romagnoli A., Keats A. S. (1980). Ceiling effect for respiratory depression by nalbuphine. *Clinical Pharmacology and Therapeutics*.

[45] Gal T. J., Difazio C. A., Moscicki J. (1982). Analgesic and respiratory depressant activity of nalbuphine: a comparison with morphine. *Anesthesiology*.

[46] Chen M. K., Chau S. W., Shen Y. C. (2014). Dose-dependent attenuation of intravenous nalbuphine on epidural morphine-induced pruritus and analgesia after cesarean delivery. *Kaohsiung Journal of Medical Science*.

[47] Fournier R., Van Gessel E., Macksay M., Gamulin Z. (2000). Onset and offset of intrathecal morphine versus nalbuphine for postoperative pain relief after total hip replacement. *Acta Anaesthesiologica Scandinavica*.

[48] Hawi A., Alcorn H., Berg J., Hines C., Hait H., Sciascia T. (2015). Pharmacokinetics of nalbuphine hydrochloride extended release tablets in hemodialysis patients with exploratory effect on pruritus. *BMC Nephrology*.

[49] Malinovsky J.-M., Lepage J.-Y., Karam G., Pinaud M. (2002). Nalbuphine reverses urinary effects of epidural morphine: a case report. *Journal of Clinical Anesthesia*.

[50] Liao C. C., Chang C. S., Tseng C. H. (2011). Efficacy of intramuscular nalbuphine versus diphenhydramine for the prevention of epidural morphine-induced pruritus after cesarean delivery. *Chang Gung Medical Journal*.

[51] Jannuzzi R. G. (2016). Nalbuphine for treatment of opioid-induced pruritus. *The Clinical Journal of Pain*.

[52] Moustafa A. A., Baaror A. S., Abdelazim I. A. (2016). Comparative study between nalbuphine and ondansetron in prevention of intrathecal morphine-induced pruritus in women undergoing cesarean section. *Anesthesia Essays and Researches*.

[53] Forbes H. J., Thomas S. L., Smeeth L. (2016). A systematic review and meta-analysis of risk factors for postherpetic neuralgia. *Pain*.

